# Untargeted Metabolomics Reveals the Protective Effect of Fufang Zhenshu Tiaozhi (FTZ) on Aging-Induced Osteoporosis in Mice

**DOI:** 10.3389/fphar.2018.01483

**Published:** 2019-01-08

**Authors:** Duosheng Luo, Jingbiao Li, Kechun Chen, Xianglu Rong, Jiao Guo

**Affiliations:** ^1^Key Unit of Modulating Liver to Treat Hyperlipemia SATCM (State Administration of Traditional Chinese Medicine), Guangdong Pharmaceutical University, Guangzhou, China; ^2^Guangdong Metabolic Disease Research Center of Integrated Chinese and Western Medicine, Guangzhou, China

**Keywords:** aging-induced osteoporosis, Fufang Zhenzhu Tiaozhi, metabolomics, ultra performance liquid chromatography, mass spectrometry

## Abstract

Fufang Zhenzhu Tiaozhi (FTZ), as an effective traditional Chinese medicine, has been prescribed for more than 20 years. It has proven clinical efficacy as a prescription for patients with dyslipidemia, glucocorticoid- and high-fat-induced osteoporosis, but its effect on osteoporosis induced by aging is still unclear. The aim of this study was to investigate the anti-osteoporosis effect of FTZ in aging mice and revealed its biochemical action mechanism using metabolomics. Model of primary osteoporosis induced by aging was established. The mice in treatment group received a therapeutic dose of oral FTZ extract once daily during the experiment. The model and control groups received the corresponding volume of oral normal saline solution. Plasma samples of all three groups were collected after 12 weeks. Clinical biochemical parameters and biomechanics were determined in the osteoporosis model induced by normal aging to evaluate anti-osteoporosis effect of FTZ. Ultra performance liquid chromatography coupled with quadrupole time-of-flight mass spectrometry (UPLC-QTOF/MS) was used to analyze metabolic changes. The changes of histomorphometric and biomechanic parameters of femurs, as well as osteoblast and osteoclast activity indicated that FTZ administration reduced the risk of osteoporosis. Partial least squares discriminant analysis (PLS-DA) score plot revealed a clear separation trend between model and controls. Moreover, PLS-DA score plot indicated the anti-osteoporosis effect of FTZ with sphingosine 1-phosphate, LPA (16:0) and arachidonic acid (AA) among key biomarkers. The pivotal pathways revealed by pathway analysis including sphingolipid metabolism, glycerophospholipid metabolism, and AA metabolism. The mechanism by which FTZ reduces the risk of primary age-related osteoporosis in mice might be related to disorders of the above-mentioned pathways. FTZ has a protective effect against osteoporosis induced by aging, which may be mediated via interference with sphingolipid, glycerophospholipid, and AA metabolisms in mice.

## Introduction

Millions of people were affected by osteoporosis, which was a degenerative bone disease characterized by decreasing bone mineral content and bone strength. Osteoporosis are especially severe in, those with pathological fractures, and women accounted for more than 70% of the total number because of the decline in hormone level associated with aging (Cotts and Cifu, 2018). Traditional Chinese medicines (TCM) or natural products have been longtime used in clinic and considered as an alternative therapy for the prevention and treatment of various diseases worldwide (Liu et al., 2013; Hao et al., 2014; Moloney, 2016; Chen D. Q. et al., 2018; Chen H. et al., 2018; Wang et al., 2018a,b). Fufang Zhenzhu Tiaozhi (FTZ), as an effective traditional Chinese medicine, has been prescribed for more than 20 years in cases of dyslipidemia and glucocorticoid-treated osteoporosis (Guo et al., 2006, 2009; Sun et al., 2016), but its effect on osteoporosis induced by aging is still unclear.

Metabolomics has been applied to establish novel therapeutic targets and provide prognostic biomarkers for the early detection and diagnosis of disease progression (Johnson et al., 2016). Recently, ultra performance liquid chromatography coupled with quadrupole time-of-flight mass spectrometry (UPLC-QTOF/MS)-based metabolomics was widely used in pharmacological bioactivity and toxicity evaluation of TCM (Chen D.Q. et al., 2016; Shi et al., 2016; Wang et al., 2017). The development and progression of postmenopausal osteoporosis were associated with many pathophysiological factors including estrogen receptor dysregulation (Gallagher and Jeffre, 2011), pro-inflammatory factors (Barbour et al., 2012), oxidative stress (Callaway and Jiang, 2015), and OPG/RANK/RANKL system (Hofbauer and Schoppet, 2004). There is still no sensitive and specific biomarker that can reflect the pathogenesis of osteoporosis. Metabolomics projects usually yield clusters of biomarkers, which make it potentially useful for the study of the development and progression of osteoporosis. Ovariectomized (OVX) animal models were the most widely used model organisms in the study of osteoporosis. By contrast, we used normal aging mice as a model in this study in conjunction with metabolomics to explore the changes of endogenous metabolites and the effects of FTZ.

In current study, we first sought to establish aging female mice as animal models of postmenopausal osteoporosis. Secondly, blood biochemical parameters, bone microarchitecture histomorphometry, and bone biomechanics were used to evaluate the characteristics of the postmenopausal osteoporosis disease in aging mice. Finally, UPLC-QTOF/MS was used to delineate the metabolic profile to identify and characterize specific metabolic pathways with potential relevance for treatment and intervention of FTZ in postmenopausal osteoporosis.

## Materials and Methods

### Chemicals and Reagents

Formic acid was purchased from Merck company (Germany). LC-grade acetonitrile was purchased from the Merck company (Germany). Ultra-high purity water was prepared using a Milli-Q water purification system. Other chemicals were of analytical grade and purity was above 99.5%. The components of FTZ, including *Citri sarcodactylis* fructus, *Ligustri lucidi* fructus, *Salviae miltiorrhizae* radix et rhizoma, *Notoginseng radix* et rhizoma, *Coptidis rhizoma*, *Atractylodis macrocephalae* rhizoma, *Cirsii japonici herba* et radix, and *Eucommiae cortex*, were obtained from Zhixin Chinese Herbal Medicine Co., Ltd. (Guangzhou, China). Professor Wei He, Guangdong Pharmaceutical University authenticated the plant material using the Pharmacopeia of the people’s republic of China identification key (ISBN 2015, volume I). The voucher specimens were GDPUZYY 20110901-8 (Supporting Material-Voucher specimen) (Guo et al., 2011). Authentic reference FTZ was provided by the Institute of Materia Medica, Guangdong Pharmaceutical University. HPLC fingerprinting was used to confirm the quality of the FTZ extract (Zhong et al., 2012), as shown in Supplementary Data Sheet S1.

### Animals and Experimental Design

Eighteen specified pathogen free (SPF) C57BL/6J Narl female mice were purchased from the same vendor were obtained from the Guangdong animal experimental medical center. The Animal Ethics Committee of Guangdong Pharmaceutical University approved the study. The mice were housed in cages at a room temperature of 25 ± 2°C, with free access to standard solid food and water. The eighteen mice were randomly divided after 1 week of acclimatization to yield a model group (21 months old), a treatment group (21 months old), and a control group (4 months old). The treatment group received 1.0 g/kg/d of oral FTZ every day for 12 weeks continuously. The model and control groups were orally administered the corresponding volume of normal saline.

### Histomorphometric Analysis Using Micro Computed Tomography

A high performance *in vivo* micro-CT SKYSCAN 1176 (Bruker, Germany) was used for quantitative and qualitative analysis of the right femurs with image field at pixel size 9 μm. CTVol (Skyscan, Germany) was used to reconstruct 3D images. The distal femoral metaphyses were analyzed within a region of 1.5 mm in length 1.0 mm below the growth plate. CTAn (Skyscan) was used to quantify the trabecular bone mineral density (Tb.BMD), trabecular thickness (Tb.Th),bone surface/bone volume (BS/BV), bone surface/tissue volume (BS/TV), bone volume/tissue volume (BV/TV), trabecular separation (Tb.Sp), trabecular number (Tb.N), and structure model index (SMI) within the region of interest. SMI was used to quantify the plate-rod characteristics of the 3D trabecular structure. Cortical bone parameters including endosteal circumference (EC), cortical bone shell thickness, cortical BMD, periosteal circumference (PC), and cross-sectional area were measured in the middle of the diaphysis of the femur.

### Measurement of Serum Indicators of Osteoblast and Osteoclast Activity

The concentrations of procollagen type-I amino-terminal propeptide (PINP), osteocalcin, osteoprotegerin (OPG), bone alkaline phosphatase (BALP), pyridinoline (PYD), and crosslinked N-telopeptide of type I collagen (NTXI) in the serum were all measured by commercial kits (CUSABIO, Wuhan, Hubei, China), according to the manufacturer’s instructions.

### Bone Biomechanics Analysis Using the Three-Point Bending Method

Right femurs stored at -20°C were thawed at room temperature and then wetted with saline. The samples were placed in the INSTRON E1000 Electrodynamic static universal testing machine (Instron, United Kingdom), for testing and analysis of biomechanical properties. The parameters were set as follows: the diameter of the indenter was 1 mm, loading speed was 2 mm/min, span (L) was 10 mm. The maximum load, breaking load, bending energy, maximum displacement, and stiffness were recorded by the acquisition computer.

### Serum Metabolomics

A 300 μL aliquot of plasma was mixed with 900 μL acetonitrile in an Eppendorf tube by vortexing for 2 min. The resulting mixture was centrifuged at 20000 × *g* and 4°C for 15 min. The clear supernatant was removed and used for UPLC-QTOF/MS analysis. Positive and negative ion modes were used for all samples.

A guard column (Waters, United States) was placed in front of an ACQUITY UPLC BEH C18 column (100 mm × 2.1 mm, 1.7 μm; Waters, United States). The injection volume was 5 μL and the column temperature was 30°C. Water with 0.1% formic acid was used as solvent A and acetonitrile as solvent B. The flow rate was 0.4 mL min^-1^. The elution gradient was as follows: 0–4 min, 98% A; 4–10 min, 98–75% A; 10–12 min, 75–50% A; 12–22 min, 50–35% A; 22–28 min, 35–15% A; 28–30 min, 15–20% A.

The UPLC system was interfaced with a AB Sciex Triple TOF 5600 mass spectrometer (AB Science, United States) with the following settings: Positive ion mode: ion spray voltage, 5500 V; ion source temperature, 550°C; decluttering potential, 80 eV; collision energy, 10 eV; collision energy spread, 20 eV; 50 psi was used for the curtain gas, ion source gas 1 and gas 2. Negative ion mode: ion spray voltage, 4500 V; ion source temperature, 550°C; decluttering potential, -100 eV; collision energy, -10 eV; collision energy spread, 20 eV; 50 psi was used for the curtain gas, ion source gas 1 and gas 2. The IDA model was used to qualitatively analyze the potential biomarkers in both positive and negative ion modes.

### Statistical Analysis

Student’s *t*-test was used for all comparisons between two groups of bone measurements, and ANOVA followed by Bonferroni’s multiple *t*-test for multiple groups. Kruskal-Wallis non-parametric analysis followed by Dunn’s multiple comparison test was used for metabolomic data. Time-course data such as body weight, were analyzed using repeated measures two-way ANOVA. Differences with *p*-values < 0.05 were considered statistically significant. The scope of the variation of the data has been expressed in terms of mean six standard deviation (SD) of mean six standard error of the mean (SEM). Prism GraphPad 6 software (GraphPad Software Inc., San Diego, CA, United States) was used to analyze significance, while log10 – transformed metabolite concentrations were used for partial least squares-discriminant analysis (PLS-DA) in SIMCA-P (version 14.0, Umetrics, Umeå, Sweden). The goodness-of-fit parameter (R^2^X) and the predictive ability parameter (Q^2^) were used to analyze the quality of the models.

## Results

### Body Weight and Serum Estradiol Levels

**FIGURE 1:**
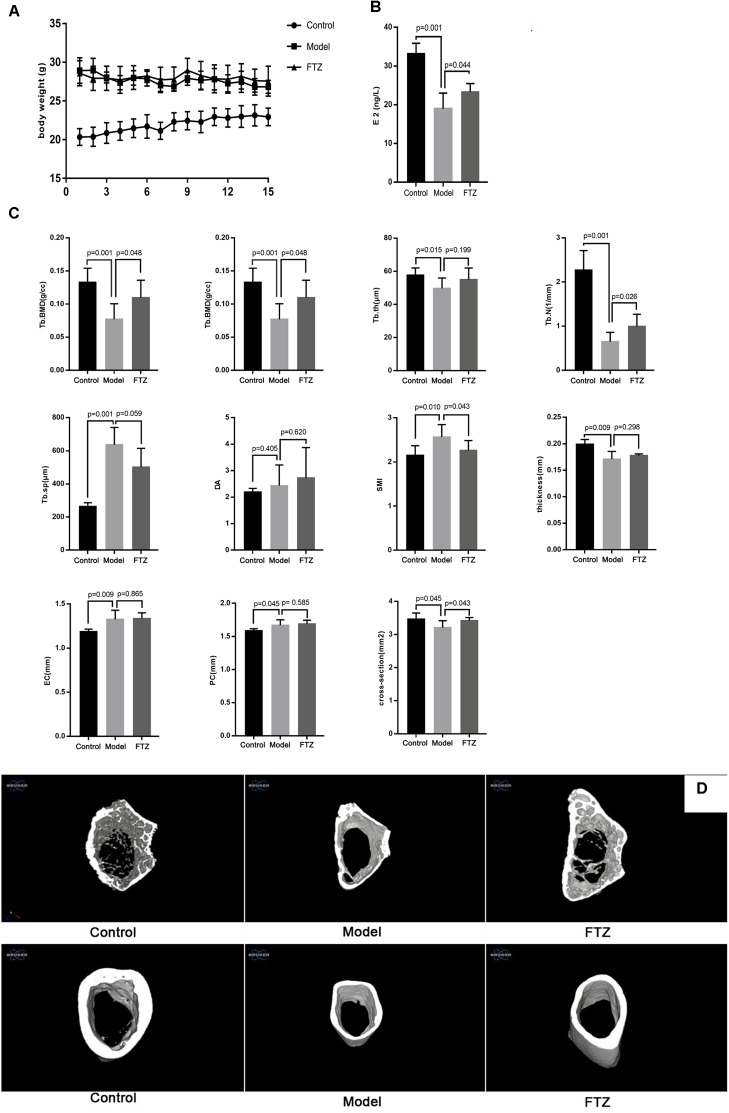
**(A)** Body weight of control, model, and FTZ groups. **(B)** Serum estradiol E2 levels of control, model, and FTZ groups. **(C)** Micro-CT femur parameters. **(D)** Micro-CT three-dimensional images. Femur images of control, model, and FTZ groups.

The average body weight for each group of mice is shown in Figure 1A. There was a significant difference between the model and control groups, but none the model group and the FTZ-treatment group. Serum E2 levels of mice in the model group were significantly decreased compared with the in control, while in the FTZ-treatment group they were significantly higher relative to the model (*p* < 0.05; Figure 1B).

### Micro Computed-Tomography Analysis

After 12 weeks, the trabecular bone parameters in the distal femur were analyzed by micro-CT (Figure 1C). There were significant decreases of Tb.BMD, BV/TV, Tb.th and Tb.N and significant increases of, Tb. Sp and SMI in model group compared with the control group, suggesting the appearance of osteoporosis symptoms. Tb. BMD, BV/TV, and Tb. N were significantly increased in the FTZ-treated mice, while Tb. Sp and SMI were significantly decreased compared with model mice. Measurements of the cortical bone in the middle of the femur showed that the model mice had a significantly thinner cortical bone structure with EC, PC, and increased cross-sections compared to the control mice. We therefore confirmed that female C57BL/6J Narl mice lost bone mass in the femur with aging. Conversely, the FTZ-treated mice did not show significant differences in EC, PC, and cross-sectional area compared with model mice.

Three-dimensional (3D) micro-CT images of the middle diaphysis of the distal femur with trabecula are shown in Figure 1D. A larger trabecular network was observed in the control mice compared to the model mice.

### Analysis of Bone Biomechanics

**FIGURE 2:**
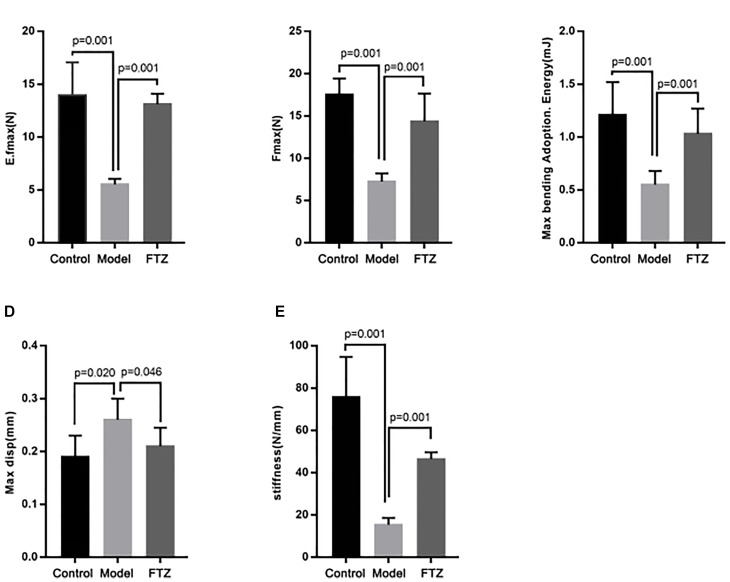
Bone biomechanics parameters of control, model, and FTZ groups. **(A–E)** Bone parameters of the distal femur analyzed by INSTRON E1000 electrodynamic static universal testing machine. **(A)** Elastic load. **(B)** Frartufe load. **(C)** Bending energy. **(D)** Displacement. **(E)** Stiffness.

After 12 weeks, the biomechanic parameters of the tibia were analyzed using a static universal testing machine (Figure 2). There were significant decreases of the elastic load, bending energy, fracture load, and stiffness, while displacement was significantly increased in model mice compared with control mice, suggesting that the biomechanical performance of the tibia deteriorated with age in the mice. Conversely, the elastic load, bending energy, fracture load, and stiffness were significantly increased, while displacement was significantly decreased in the FTZ-treated mice compared with model mice. Additionally, femoral fracture characterized by segment rule pulverization was mainly observed in the aged bones.

### Serum Concentrations of Markers Related to Osteoblast and Osteoclast Activity

**FIGURE 3:**
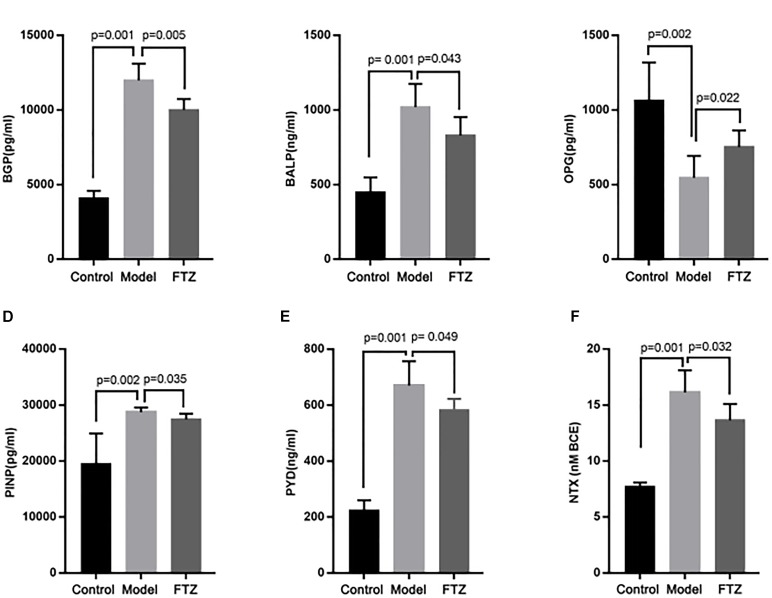
The concentrations of osteoblast and osteoclast indicators. Effects on serum osteoblast and osteoclast indicators, calcium and phosphate content ratio of control, model, and FTZ groups **(A–D)**. **(A)** BGP, osteocalcin. **(B)** ALP, alkaline phosphatase. **(C)** OPG, osteoprotegerin. **(D)** PINP, procollagen type I amino-terminal propeptide. **(E,F)** Osteoclast indicators. **(E)** PYD, pyridinoline. **(F)** NTX, cross-linked N-telopeptide of type I collagen.

The activity of osteoblasts and osteoclasts was measured using specific markers as proxy (Figure 3). Accordingly, elevated concentrations of BALP, osteocalcin, BGP, and PINP indicate osteoblast activity, whereas elevated concentrations of PYD and NTXI indicate osteoclast activity. The serum concentrations of osteocalcin, BALP, and were significantly increased PINP in the model mice, whereas serum OPG showed a significant decrease compared with the control mice. Serum PYD and NTXI were significantly increased in the model mice. These results suggest the presence of age-related osteoporosis of the high-conversion type, whereby both bone formation and resorption were increased, but the increase of bone resorption was more pronounced. There were decreased in FTZ-treatment mice compared with model mice.

### Serum Metabolomics

**FIGURE 4:**
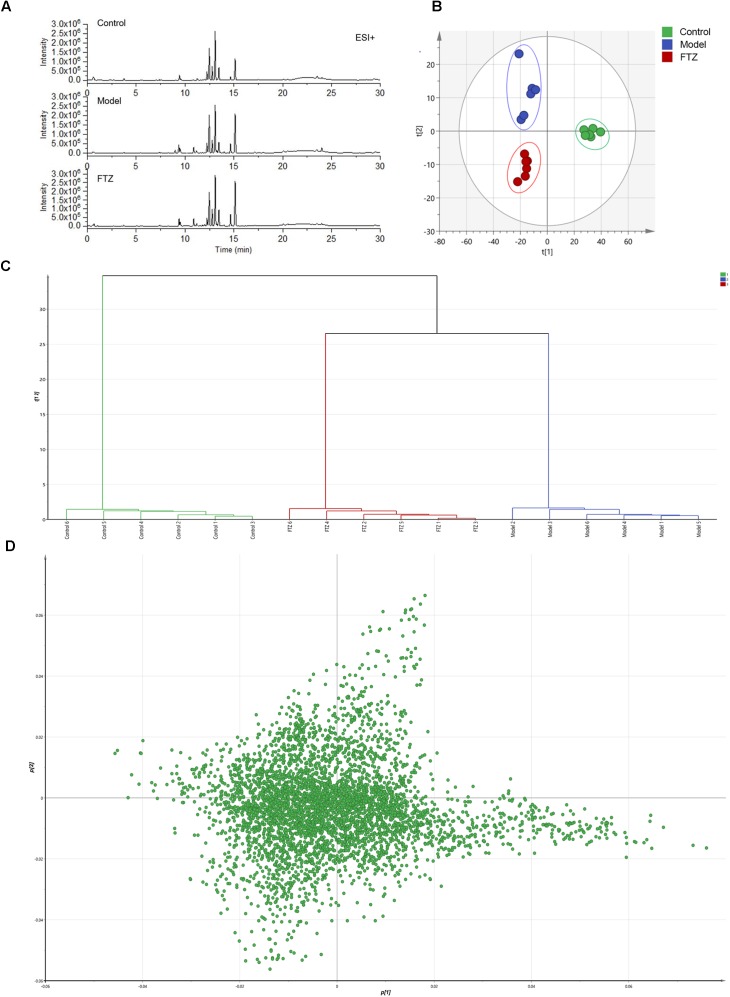
Metabolic profiling and multivariate statistical analysis. **(A)** Base peak intensity chromatograms of control, model, and FTZ groups in positive ion mode obtained from UPLC-QTOF/MS analysis. **(B)** Score plot of PLS-DA of control, model, and FTZ groups. [*R*^2^X(cum) = 0.971, *Q*^2^(cum) = 0.738]. **(C)** Clustering analysis of control, model, and FTZ groups. **(D)** Loading plot of PLS-DA in positive ion mode from control, model, and FTZ groups.

The repeatability and precision were tested by six reduplicate analyses from the QC samples and six samples, respectively. RSD% of retention time and peak area were below 0.16 and 4.83%, respectively. The data showed that the repeatability and precision meet the demand of this study. As can be seen in the typical base peak intensity chromatograms of the control, model, and FTZ-treated groups (Figure 4A), the UPLC–QTOF/MS demonstrated good reproducibility. The influence of FTZ on the metabolite patterns in the plasma was investigated using the PLS-DA model. The resulting projection model provided the latent variables (LVs) with a focus on maximum separation. Score plots of the PLS-DA model [ESI^+^: *R*^2^X(cum) = 0.971, *Q*^2^(cum) = 0.738] are shown in Figure 4B. The permutation test have done[*R*^2^ = (0.0,0.168), *Q*^2^ = (0.0,-0.267)], which implied a high quality of the PLS-DA model. The control, model and FTZ groups were significantly separated, whereby the samples from the model group had the opposite direction along LV2 compared with those of the control group. By contrast, control group samples had the opposite direction along LV1 compared with the FTZ and model groups. The clustering analysis revealed that the FTZ-treatment group was located between the model and control groups (Figure 4C). The results therefore support the efficacy of FTZ in mitigating age-related osteoporosis.

The significantly altered metabolites were searched by selecting variables on the basis of the VIP values in the loading plot of the PLS-DA model (Figure 4D). To identify the metabolites that contributed most to the clustering, the variables with importance values above 1.5 and *p*-values of independent Student’s *t*-test between two groups <0.05 were selected. The thus endogenous metabolites were subjected to further structural identification according to the previous reported methods (Zhao et al., 2013; Chen H. et al., 2016; Chen et al., 2017). The resulting potential biomarkers are listed in Table 1. Structures corresponding to the selected metabolites were obtained by searching the following databases: HMDB^1^, LIPID MAPS^2^, METLIN^3^, and KEGG^4^. Finally, 12 differentially abundant metabolites were identified. These included neuroprotectin D1, fructose 1,6-bisphosphate, NADH, arachidonic acid (AA), lysophosphatidylcholines (LysoPCs), glycocholic acid, taurodeoxycholic acid, LPA (16:0), lysoPE (22:5), DG (36:3), and sphingosine 1-phosphate (S1P). The fold changes (FC) based on the relative intensities and biological corresponding pathways are listed in Table 1.

**Table 1:** Identified potential biomarkers, fold changes (FC) and *p*-values among control, model, and FTZ groups.

No.	Metabolite	t_R_–m/z	Elemental composition	Model vs. Control		FTZ vs. Model		FTZ vs. Control	
						
				FC	*P*-value	FC	*P*-value	FC	*P*-value
1	Sphingosine 1-phosphate	20.52–380.2090	C_18_H_38_NO_5_P	6.58	0.00	0.84	0.02	5.50	0.00
2	LPA (0:0/16:0)	11.95–452.2801	C_19_H_39_O_7_P	0.32	0.00	1.53	0.00	0.49	0.00
3	LysoPE (22:5)	12.73–528.3105	C_27_H_46_NO_7_P	0.70	0.02	0.64	0.00	0.45	0.00
4	DG (36:3)	16.11–619.4063	C_39_H_7_O_5_	0.01	0.00	1.75	0.00	0.03	0.00
5	PC (16:0/18:1)	28.43–802.5384	C_46_H_76_NO_8_P	0.63	0.01	1.37	0.01	0.86	0.21
6	PC (40:9)	20.52–828.5545	C_48_H_78_NO_8_P	1.60	0.00	1.27	0.03	2.02	0.00
7	Neuroprotectin D1	17.64–361.1682	C_22_H_32_O_4_	1.47	0.00	1.24	0.04	1.84	0.00
8	Arachidonic acid	8.98–305.1617	C_20_H_32_O_2_	3.78	0.00	0.68	0.00	2.58	0.00
9	Fructose 1,6-bisphosphate	15.93–341.1827	C_6_H_14_O_12_P_2_	16.79	0.00	0.01	0.00	0.20	0.00
10	NADH	29.16–666.3661	C_21_H_29_N_7_O_14_P_2_	0.20	0.00	0.49	0.00	0.10	0.00
11	Glycocholic acid	15.73–466.3327	C_26_H_43_NO_6_	1.24	0.04	0.28	0.00	0.35	0.00
12	Taurodeoxycholic acid	11.73–500.2791	C_26_H_45_NO_6_S	0.41	0.00	0.39	0.00	0.16	0.00


*VIP values were obtained from the model. FC was calculated based on a binary logarithm for model vs. control and FTZ vs. model. FC with a value greater than zero indicates a higher intensity of the plasma metabolite, while a FC value less than zero indicates a lower intensity of the plasma metabolite. *P*-values are calculated by one-way ANOVA.*

**FIGURE 5:**
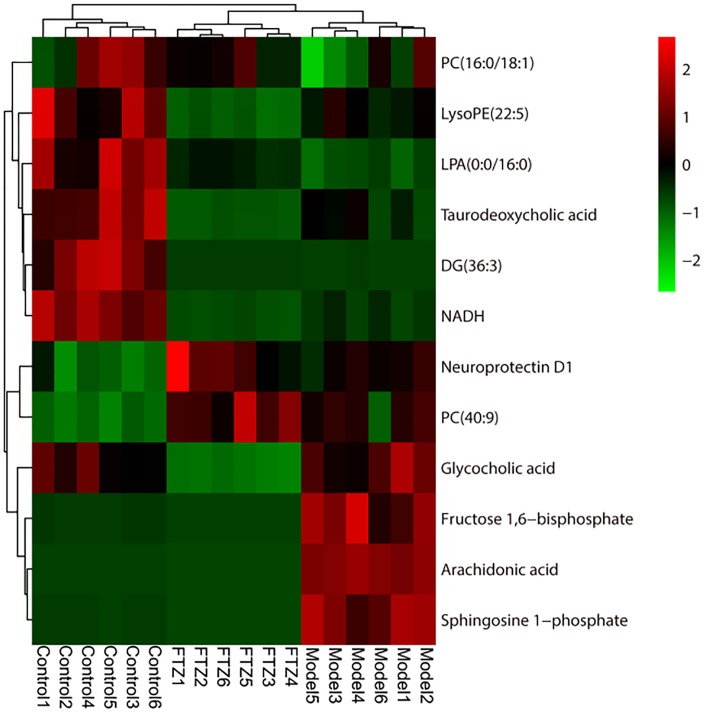
Heatmap of 12 potential biomarkers among control, model, and FTZ groups. Red and green indicate increased and decreased levels, respectively.

The results of the heatmap analysis indicated that samples in the FTZ-treatment group were similar to the control (Figure 5). By contrast, the PLS-DA score plots revealed a clear difference between the model and FTZ-treatment groups, implying that FTZ significantly improved the metabolic profile in the plasma of osteoporotic mice.

**FIGURE 6:**
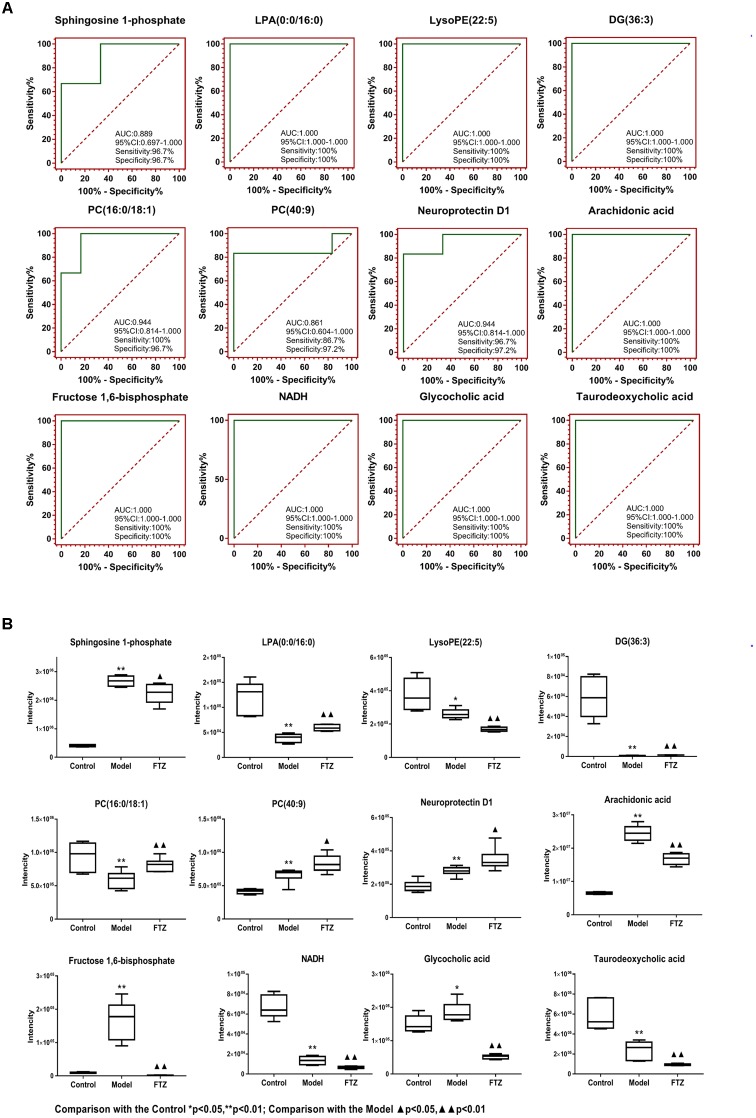
**(A)** PLS-DA-Based ROC curves of the 12 potential biomarker of anti-osteoporosis effects of FTZ. The associated AUC, 95% CI, sensitivities, and specificities are indicated. **(B)** Relative intensity analysis of 12 potential biomarkers. Box plots showing significant changes in the levels of 12 potential biomarkers among the control, model, and FTZ groups. The statistical significance between the two groups is marked; ^∗^*p* < 0.05, ^∗∗^*p* < 0.01 significant difference compared with control group; ^
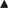
^*p* < 0.05, ^
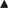

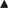
^*p* < 0.01 significant difference compared with model group. *Y*-axis: normalized relative intensity.

Potential biomarkers of the anti-osteoporosis effects of FTZ were searched using PLS-DA-based ROC curves. The 12 biomarkers shown in Figure 6A had high sensitivity (>90%), specificity (>90%), and AUC values (>0.80), which makes them potential biomarkers for the anti-osteoporosis effect of FTZ. Figure 6B illustrates the difference in the level of the potential biomarkers among the control group and model group and FTZ-treated groups. These results indicated that Sphingosine 1-phosphate, LPA (16:0), AA represent potential biomarkers of the anti-osteoporosis effects of FTZ.

**FIGURE 7:**
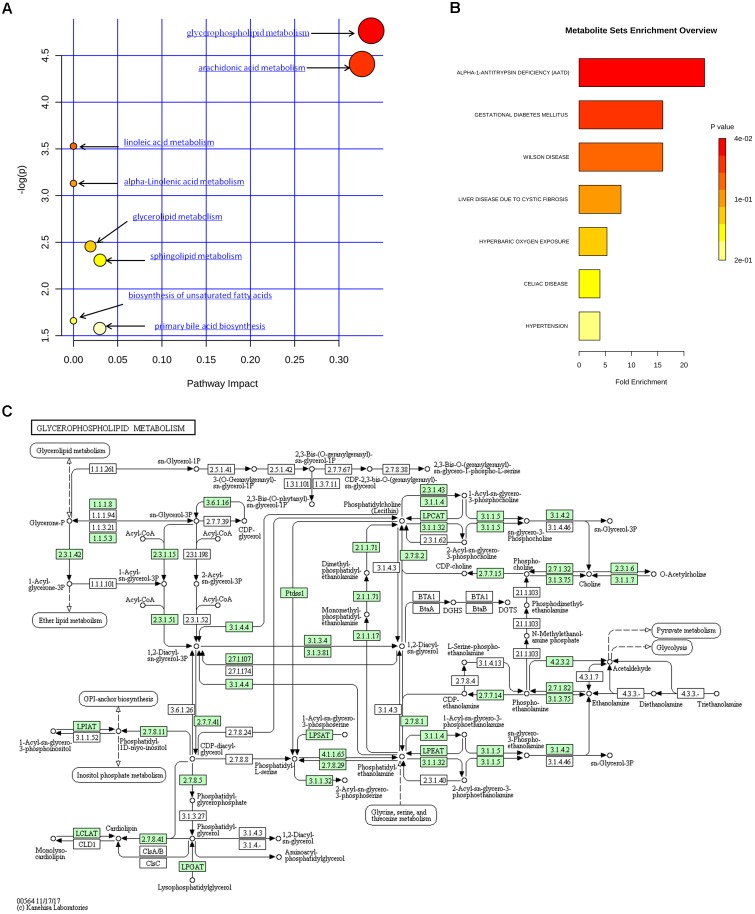
Metabolic pathway analysis of identified differential species. **(A)** Summary of IPA with MetPA. **(B)** QEA performed using MSEA. **(C)** Overview of glycerophospholipid metabolism with MetPA (reference map by KEGG). Green boxes represent enzymatic activities with putative cases of analogy in mice.

The metabolic pathways and networks possibly influenced by aging were searched via MetPA analysis. IPA showed dysregulation in eight pathways, including the metabolism of sphingolipids, glycerolipids, glycerophospholipids, biosynthesis of unsaturated fatty acids, and primary bile acids, as well as the metabolism of arachidonic-, linoleic-, and alpha-linolenic acid in the mice with age-induced osteoporosis (Figure 7A and Table 2). An example of the results of biological pathway analysis is illustrated with glycerophospholipid metabolism as an example (Figure 7C). The effects obtained for the other pathways are shown in Supplementary Figures S1–S7. In addition, the QEA algorithm of the MSEA method revealed the dysregulation of seven metabolic pathways in age-induced osteoporosis (Figure 7B).

**Table 2:** Ingenuity pathway analysis with MetPA from differential biomarkers.

Pathway name	Total metabolites	Hits	*p*	-log(*p*)	Holm *p*	FDR	Impact	Details
Glycerophospholipid metabolism	30	2	0.0085	4.76	0.70	0.50	0.336	Figure 7C
Arachidonic acid metabolism	36	2	0.0122	4.41	0.98	0.49	0.326	Supplementary Figure S1
Linoleic acid metabolism	6	1	0.0293	3.53	1.00	0.80	0.000	Supplementary Figure S2
Alpha-Linolenic acid metabolism	9	1	0.0437	3.13	1.00	0.89	0.000	Supplementary Figure S3
Glycerolipid metabolism	18	1	0.0858	2.46	1.00	1.00	0.019	Supplementary Figure S4
Sphingolipid metabolism	21	1	0.0994	2.31	1.00	1.00	0.030	Supplementary Figure S5
Biosynthesis of unsaturated fatty acids	42	1	0.1903	1.66	1.00	1.00	0.000	Supplementary Figure S6
Primary bile acid biosynthesis	46	1	0.2067	1.58	1.00	1.00	0.029	Supplementary Figure S7


*A “total” is the total number of differential metabolites species in the pathway; “hits” is the actually matched number from the user differential biomarkers; the raw p is the original p calculated from the enrichment analysis; the “Holm p” is the *p*-value adjusted by Holm-Bonferroni method; “impact” is the pathway impact value calculated from pathway topology analysis.*

## Discussion

The three types of primary osteoporosis are postmenopausal osteoporosis (type I), senile osteoporosis (type II), and idiopathic osteoporosis. Aging women account for the majority of patients due to the double impact of age and declining hormone levels. Type 1 osteoporosis is characterized by reduced hormone levels, which results in accelerated bone turnover leading to bone loss. The earliest OVX rat and mouse models were established in 1991 (Kalu, 1991). These models revolutionized the study of bone loss because the removal of the ovaries mimics postmenopausal hormonal changes that occurs in humans, including in the decreased estrogen levels (Caverzasio et al., 2008; Guillerminet et al., 2010). Nevertheless, these models still have some disadvantages. Firstly, the postmenopausal model relies on ovariectomy, which does reflect the decrease of estrogen levels, but does not reflect practical and clinical discrepancies. While ovarian dysfunction indeed plays a role in osteoporosis, the ovarian stromal cells in postmenopausal women still retain some endocrine function rather than completely disappearing. In addition, the surgical intervention induces physical stress, resulting in inconsistent laboratory values (Giangregorio and Blimkie, 2002).

In this study, we established a model of osteoporosis based on normal aging female mice. The weight of the aging mice over 24 months of age was increased compared to young 7 months old mice, and there was no significant difference between the model group and the FTZ-treatment group. This was in agreement with previous studies which reported that body weight was increased in the ovariectomized mouse model of osteoporosis (Zhang et al., 2009; Zhao et al., 2011; Kim et al., 2016).

Several serum indicators of osteoblast and osteoclast activity were examined. The aging mice exhibited higher levels of the osteoblast markers BALP, osteocalcin and PINP, but lower OPG. Similarly, Liao et al. (2002) observed that serum BALP increases with age, while Lofman et al. (2005) reported that serum osteocalcin increased in postmenopausal women, with continuously increased levels for about 15 years, which was significantly different compared with pre-menopausal women. Reginster et al. (2004) reported that the serum levels of PINP decreased among 7705 postmenopausal women that were treated with raloxifene, which supports the observations presented here. Furthermore, the aging mice exhibited higher levels of the osteoclast markers NTXI and PYD, which corroborated the characteristically high bone turnover rate indicative of osteoporosis. Similarly, Iwamoto et al. (2007) observed that NTX levels were significantly increased in osteoporotic postmenopausal women compared to normal premenopausal controls. Chopin et al. (2012) observed a significant negative correlation between PYD and BMD in postmenopausal women, whereby the PYD content was significantly higher than in pre-menopausal control. In this study, the FTZ-treated group exhibited lower NTXI and PYD, which suggested that FTZ may decrease bone resorption.

We evaluated the age-related changes in trabecular architecture in C57BL/6J mice, which showed that the trabecular BV/TV decreased significantly. Moreover, the decrease of Tb.N and Tb.BMD, indicated trabecular bone loss. By contrast, Tb.Sp and SMI increased compared with the control mice, which was consistent with previous reports on the ovariectomized model (Liao et al., 2002; Ju et al., 2015). Conversely, the Tb.BMD, BV/TV, and Tb.N increased in the FTZ-treatment group, while Tb.Sp and SMI decreased compared with the model group, which indicates that FTZ may increase bone mass and strength.

We studied the age-related deterioration of the tibiae of C57BL/6J female mice, which showed that elastic load, fracture load, bending energy, and stiffness decreased significantly compared with the control group. Conversely, the elastic load, fracture load, bending energy, and stiffness increased in the in FTZ-treatment group, which suggested that FTZ has a positive effect on the biomechanical properties of bones.

Metabolomics can measure the changes in the levels of metabolites found in biological fluids and tissues, yielding a quantitative and comprehensive picture of metabolic pathway activity (DeBerardinis and Thompson, 2012). In the previous metabolomics study on osteoporosis, OVX animal models were used usually Chen et al. (2014). Chen et al. (2014) used OVX rats and found significantly higher concentrations of metabolites associated with energy metabolism, including TCA-cycle intermediates, glucose, fumarate, and taurine, as well as lower concentrations of 3-hydroxybutyrate, allantoin, citrate, malate, succinate, aspartate, glycine, acetate, ethanol, and dimethylamine rats as osteoporosis model and found that OVX group significantly higher concentrations of metabolites associated with energy metabolism (including TCA-cycle intermediates and glucose), fumarate, taurine. Lower concentrations of 3-hydroxybutyrate, allantoin, citrate, malate, succinate, aspartate, glycine, acetate, allantoin ethanol, and dimethylamine. The metabolomic analyses of rat plasma in the study of Ma et al. (2013) showed that levels of AA, octadecadienoic acid, branched-chain amino acids (valine, leucine, and isoleucine), homocysteine, hydroxyproline, and ketone bodies (3-hydroxybutyric acid) significantly elevated, while levels of docosahexaenoic acid, dodecanoic acid, and lysine significantly decreased in OVX group compared with those in the homeochronous sham group. In this study, we also observed metabolic differences between the model and control groups. One interesting class of differential metabolites was phospholipids.

Phospholipids are a class of lipids containing phosphoric acid, with glycerophospholipids and sphingomyelin components being the most prominent (Blackburn and Mansell, 2012). Lysophosphatidic acid (LPA) serves in the phospholipid metabolism not only as an intermediate, but also as cellular messenger. It can promote proliferation, growth, differentiation and other important biological behaviors (Gennero et al., 2011). Increasing evidence suggests that osteoblasts can produce LPA which then acts as an autocrine signaling molecule in the bone microenvironment that can also serve as a paracrine signaling molecule, with a profound effect on bone development (Salles et al., 2013). LPA was a potential mediator of osteoblast-osteoclast signaling in bone (Gennero et al., 2011; Salles et al., 2013; Sims et al., 2013; Marion et al., 2014). The regulatory effect of LPA on osteoblasts and osteoclasts is mediated by P2X7 nucleotide receptor signaling which can produce LPA, revealing a new signaling axis that may play a key role in bone mechanical conduction. LPA receptor subtypes have multiple effects on mature bone cells, including cell contraction and modulation of calcium ion concentration, activation of the key transcription factor NFATc1, promoting osteoclast survival. LPA also promotes the integration of osteoclasts, which leads to the formation of larger cells, and increased numbers of osteoclasts have been found in pathological bone resorption. Thus, LPA and its receptors play an important role in the pathophysiology of osteoporosis (Sims et al., 2013). Phosphatidylcholines (PC) are produced by phospholipase A2, which is activated under inflammatory conditions. It hydrolyzes lysophosphatidylcholine (LPC), which is a bioactive pro-inflammatory lipid generated through pathological activities and a clinical diagnostic indicator of certain pathophysiological states (Matsumoto et al., 2007, Matsumoto), including ovariectomy-induced osteoporosis (Allard-Chamard et al., 2014). Moreover, Liu et al. (2012) reported that the LPC levels increased significantly in the plasma of osteoporotic rats. In these animals, the production of reactive oxygen species (ROS) may bring about extensive bone loss and skeletal fragility, resulting in acute osteoporosis.

In the study found that S1P was one of the potential biomarkers for aged mice with primary osteoporosis compare with young group. S1P Previous studies had confirmed that S1P plays an important role in bone metabolism and become a new therapeutic strategy for the treatment of osteoporosis by regulating osteoclast-related bone remodeling (Ishii et al., 2009; Junichi et al., 2013; Kim et al., 2016; Weske et al., 2018). S1P is an important factor in the communication between bone resorption and bone formation, its effect on bone metabolism in humans depends on its plasma/medullary gradient.

S1P can control the trafficking of osteoclast precursors between bone marrow and the circulation cavities via the S1PR G protein-coupled receptors. When the bone-marrow concentration of S1P is low, S1PR1 acts as a chemoattractant for S1P in the bone marrow. By contrast, when the S1P concentration in the blood is high, S1PR2 mediates chemorepulsion. The regulation of precursor recruitment may yield novel therapeutic strategies for the control osteoclast-dependent bone remodeling (Maceyka et al., 2012; Ishii and Kikuta, 2013).

The fatty acid levels were significantly perturbed in the process of bone loss, which was particularly reflected in our metabolomic data. AA is released from membrane phospholipids by phospholipase hydrolysis of unsaturated fatty acids. It was found to promote osteoclast genesis by stimulating RANK-L expression and inhibiting OPG secretion in osteoblasts (Casado-Díaz et al., 2013). Moreover, its metabolites such as epoxyeicosatrienoic acids (EETs), which include prostaglandins and leukotrienes, were recently shown to affect osteoclasts and bone loss. EETs significantly increased bone loss and inhibited osteoclast formation and activity, and reduced NF-κB ligand (RANKL) receptor activation (Guan et al., 2015). Moreover, leukotrienes have also been implicated in bone remodeling, specifically in osteoporosis and rheumatoid arthritis (Abrahamsen et al., 2000; Tilg et al., 2008). The metabolic pathway of AA might act as an important factor in the pathogenesis of osteoporosis according to our study data, since it is perturbed by aging.

Generally, estrogen deficiency associated with aging is the main cause of postmenopausal osteoporosis. In contrast with previous studies that used the OVX model, we used aging female mice as a more natural osteoporosis model to study aging-induced bone loss, investigate the characteristic parameters of osteoporosis and obtain metabolomic profiles. On the basis of the current results, we identified potential biomarkers with important biological significance. Most importantly, FTZ was found to reverse the abnormal levels of metabolites related to the metabolism of phospholipids, AA and energy. These alterations of endogenous metabolites confirmed that the protective effect of FTZ against osteoporosis was systemic.

## Conclusion

A metabolomic approach based on UPLC-QTOF/MS and multivariate statistical analysis was successfully applied to investigate the protective effects of FTZ against aging-induced osteoporosis in mice. The PLS-DA score plot showed a complete separation of the model group from the FTZ-treatment group. Furthermore, the levels of phospholipids and AA in the FTZ treated group were brought back to normal levels when compared with those of the model group, which may be helpful in explaining the anti-osteoporosis effects of FTZ. At the same time, clinical biochemistry, and biomechanical analysis also validated the protective effects of FTZ against osteoporosis. Our study demonstrated that the approach based on metabolomics UPLC-QTOF/MS and multivariate analysis could reflect the systemic regulation mechanism of TCM, which makes it a promising tool for evaluating the efficacy of traditional Chinese therapies. In addition, the osteoporosis model induced by aging in mice can be used for broader osteoporosis research.

## Author Contributions

JG, XR, and DL were responsible for the conception and design of the study. DL, JL, and KC contributed to the data collection, analysis, and image processing and wrote the manuscript. All authors read and approved the final manuscript.

## Conflict of Interest Statement

The authors declare that the research was conducted in the absence of any commercial or financial relationships that could be construed as a potential conflict of interest.

## Footnotes

1 http://www.hmdb.ca/

2 http://www.lipidmaps.org

3 http://metlin.scripps.edu/

4 http://www.genome.jp/kegg/

## Supplementary Material

FIGURE S1 Overview of arachidonic acid metabolism with MetPA (reference map by KEGG). Green boxes represent enzymatic activities with putative cases of analogy in mice.

FIGURE S2 Overview of linoleic acid metabolism with MetPA (reference map by KEGG). Green boxes represent enzymatic activities with putative cases of analogy in mice.

FIGURE S3 Overview of alpha-linolenic acid metabolism with MetPA (reference map by KEGG). Green boxes represent enzymatic activities with putative cases of analogy in mice.

FIGURE S4 Overview of glycerolipid metabolism with MetPA (reference map by KEGG). Green boxes represent enzymatic activities with putative cases of analogy in mice.

FIGURE S5 Overview of sphingolipid metabolism with MetPA (reference map by KEGG). Green boxes represent enzymatic activities with putative cases of analogy in mice.

FIGURE S6 Overview of biosynthesis of unsaturated fatty acids with MetPA (reference map by KEGG). Green boxes represent enzymatic activities with putative cases of analogy in mice.

FIGURE S7 Overview of primary bile acid biosynthesis with MetPA (reference map by KEGG). Green boxes represent enzymatic activities with putative cases of analogy in mice.

DATA SHEET S1 Analytical system and chromatographic conditions of FTZ by HPLC.

## References

[B1] Abrahamsen B. Bonnevie-Nielsen V. Ebbesen E. N. Gram J. Beck-Nielsen H. (2000). Cytokines and bone loss in a 5-year longitudinal study–hormone replacement therapy suppresses serum soluble interleukin-6 receptor and increases interleukin-1-receptor antagonist: the danish osteoporosis prevention study. *J. Bone Miner. Res.* 15 1545–1554. 10.1359/jbmr.2000.15.8.1545 10934653

[B2] Allard-Chamard H. Dufort P. Haroun S. de Brum-Fernandes A. J. (2014). Cytosolic phospholipase A2 and eicosanoids modulate life, death and function of human osteoclasts in vitro. *Prostaglandins Leukot. Essent. Fatty Acids.* 90 117–213. 10.1016/j.plefa.2013.12.009 24508380

[B3] Barbour K. E. Boudreau R. Danielson M. E. Youk A. O. Wactawski-Wende J. Greep N. C. (2012). Inflammatory markers and the risk of hip fracture: the women’s health initiative. *J. Bone Miner. Res.* 27 1167–1176. 10.1002/jbmr.1559 22392817 PMC3361578

[B4] Blackburn J. Mansell J. P. (2012). The emerging role of lysophosphatidic acid (LPA) in skeletal biology. *Bone* 50 756–762. 10.1016/j.bone.2011.12.002 22193551

[B5] Callaway D. A. Jiang J. X. (2015). Reactive oxygen species and oxidative stress in osteoclastogenesis, skeletal aging and bone diseases. *J. Bone Miner. Metab.* 33 359–370. 10.1007/s00774-015-0656-4 25804315

[B6] Casado-Díaz A. Santiago-Mora R. Dorado G. Quesada-Gómez J. M. (2013). The omega-6 arachidonic fatty acid, but not the omega-3 fatty acids, inhibits osteoblastogenesis and induces adipogenesis of human mesenchymal stem cells: potential implication in osteoporosis. *Osteoporos. Int.* 24 1647–1661. 10.1007/s00198-012-2138-z 23104199

[B7] Caverzasio J. Higgins L. Ammann P. (2008). Prevention of trabecular bone loss induced by estrogen deficiency by a selective p38 alpha inhibitor. *J. Bone. Miner. Res.* 23 1389–1397. 10.1359/jbmr.080410 18442314

[B8] Chen D. Q. Cao G. Chen H. Liu D. Su W. Yu X. Y. (2017). Gene and protein expressions and metabolomics exhibit activated redox signaling and wnt/β-catenin pathway are associated with metabolite dysfunction in patients with chronic kidney disease. *Redox Biol.* 12 505–521. 10.1016/j.redox.2017.03.017 28343144 PMC5369369

[B9] Chen D. Q. Chen H. Chen L. Tang D. D. (2016). Metabolomic application in toxicity evaluation and toxicological biomarker identification of natural product. *Chem. Biol. Interact.* 252 114–130. 10.1016/j.cbi.2016.03 27041073

[B10] Chen D. Q. Feng Y. L. Cao G. (2018). Natural products as a source for antifibrosis therapy. *Trends Pharmacol. Sci.* 39 937–952. 10.1016/j.tips.2018.09.002 30268571

[B11] Chen H. Cao G. Chen D. Q. Wang M. Vaziri N. D. Zhang Z. H. (2016). Metabolomics insights into activated redox signaling and lipid metabolism dysfunction in chronic kidney disease progression. *Redox Biol.* 10 168–178. 10.1016/j.redox.2016.09.014 27750081 PMC5066525

[B12] Chen H. Yang T. Wang M. C. (2018). Novel RAS inhibitor 25-O-methylalisol F attenuates epithelial-to-mesenchymal transition and tubulo-interstitial fibrosis by selectively inhibiting TGF-β-mediated Smad3 phosphorylation. *Phytomedicine* 42 207–218. 10.1016/j.phymed.2018.03.034 29655688

[B13] Chen S. Y. Yu H. T. Kao J. P. Yang C. C. Chiang S. S. Mishchuk D. O. (2014). An NMR metabolomic study on the effect of alendronate in ovariectomized mice. *PLoS One* 9:e106559. 10.1371/journal.pone.0106559 25184758 PMC4153652

[B14] Chopin F. Biver E. Funck-Brentano T. Bouvard B. Coiffier G. Garnero P. (2012). Prognostic interest of bone turnover markers in the management of postmenopausal osteoporosis. *Joint Bone Spine* 79 26–31. 10.1016/j.jbspin.2011.05.004 21723772

[B15] Cotts K. G. Cifu A. S. (2018). Treatment of osteoporosis. *JAMA* 10 1040–1041. 10.1001/jama.2017.21995 29536084

[B16] DeBerardinis R. J. Thompson C. B. (2012). Cellular metabolism and disease: what do metabolic outliers teach us? *Cell* 148 1132–1144. 10.1016/j.cell.2012.02.032 22424225 PMC3337773

[B17] Gallagher J. C. Jeffrey P. L. (2011). Preventing osteoporosis in symptomatic postmenopausal women. *Menopause* 18 109–118. 10.1097/gme.0b013e3181e324a6 20661164

[B18] Gennero I. Laurencin-Dalicieux S. Conte-Auriol F. Briand-Mésange F. Laurencin D. Rue J. (2011). Absence of the lysophosphatidic acid receptor LPA1 results in abnormal bone development and decreased bone mass. *Bone* 49 395–403. 10.1016/j.bone.2011.04.018 21569876 PMC3697734

[B19] Giangregorio L. Blimkie C. J. (2002). Skeletal adaptations to alterations in weight-bearing activity: a comparison of models of disuse osteoporosis. *Sports Med.* 32 459–476. 10.2165/00007256-200232070-00005 12015807

[B20] Guan H. Zhao L. Cao H. Chen A. Xiao J. (2015). Epoxyeicosanoids suppress osteoclastogenesis andprevent ovariectomy-induced bone loss. *FASEB J.* 29 1092–1101. 10.1096/fj.14-262055 25466887

[B21] Guillerminet F. Beaupied H. Fabien-Soulé V. Tomé D. Benhamou C. L. Roux C. Blais A. (2010). Hydrolyzed collagen improves bone metabolism and biomechanical parameters in ovariectomized mice: an in vitro and in vivo study. *Bone* 46 827–834. 10.1016/j.bone.2009.10.035 19895915

[B22] Guo J. Bei W. J. Tang C. P. (2009). The effect of fufang zhenshu tiaozhi extract on hepatic lipase in diet-induced hyperlipidemic rats. *Zhong Yao Cai* 32 582–585.

[B23] Guo J. Bei W. J. Hu Y. M. Tang C. P. He W. Liu X. B. (2011). A new TCM formula FTZ lowers serum cholesterol by regulating HMG-CoA reductase and CYP7A1 in hyperlipidemic rats. *J. Ethnopharmacol.* 135 299–307. 10.1016/j.jep.2011.03.012 21396994

[B24] Guo T. Guo J. Liang Y. Y. (2006). The effect of fufang zhenshu tiaozhi on the viscosity of plasma and whole blood in hyperlipidemic patients. *J. Pract. Tradit. Chin. Med.* 22 608–609.

[B25] Hao H. Zheng X. Wang G. (2014). Insights into drug discovery from natural medicines using reverse pharmacokinetics. *Trends Pharmacol. Sci.* 35 168–177. 10.1016/j.tips.2014.02.001 24582872

[B26] Hofbauer L. C. Schoppet M. (2004). Clinical implications ofthe osteoprotegerin/RANKL/RANK system for bone and vascular diseases. *JAMA* 292 490–495. 10.1001/jama.292.4.490 15280347

[B27] Ishii M. Egen J. G. Klauschen F. Meier-Schellersheim M. Saeki Y. Vacher J. (2009). Germain.sphingosine-1-phosphate mobilizes osteoclast precursors andregulates bone homeostasis. *Nature* 7237 524–528. 10.1038/nature07713 19204730 PMC2785034

[B28] Ishii M. Kikuta J. (2013). Sphingosine-1-phosphate signaling controlling osteoclasts and bone homeostasis. *Biochim. Biophys. Acta* 1831 223–227. 10.1016/j.bbalip.2012.06.002 22691949

[B29] Iwamoto J. Takeda T. Sato Y. Uzawa M. (2007). Comparison of the effect of alendronateon lumbar bone mineral density and bone turnover in men andpostmenopausal women with osteoporosis. *Clin. Rheumatol.* 26 161–167. 10.1007/s10067-006-0252-z 16565894

[B30] Johnson C. H. Ivanisevic J. Siuzdak G. (2016). Metabolomics: beyond biomarkers and towards mechanisms. *Nat. Rev. Mol. Cell Biol.* 17 451–459. 10.1038/nrm.2016.25 26979502 PMC5729912

[B31] Ju Y. I. Sone T. Ohnaru K. Tanaka K. Fukunaga M. (2015). Effect of swimming exercise on three-dimensional trabecular bone microarchitecture in ovariectomized rats. *J. Appl. Physiol.* 119 990–997. 10.1152/japplphysiol.00147.2015 26338454

[B32] Junichi K. Kawamura S. Okiji F. Shirazaki M. Sakai S. Saito H. (2013). Sphingosine-1-phosphate-mediated osteoclastprecursor monocyte migration is a critical point ofcontrol in antibone-resorptive action of active vitamin D. *PNAS* 17 7009–7013 23569273 10.1073/pnas.1218799110 PMC3637769

[B33] Kalu D. N. (1991). The ovariectomized rat model of postmenopausal boneloss. *Bone Miner.* 15 175–191. 10.1016/0169-6009(91)90124-I 1773131

[B34] Kim B.-J. Shin K.-O. Kim H. Ahn S. H. Lee S. H. Seo C.-H. (2016). The effect of sphingosine-1-phosphate on bone metabolism in humans depends on its plasma/bone marrow gradient. *J. Endocrinol. Invest.* 39 297–303. 10.1007/s40618-015-0364-x 26219613

[B35] Liao E. Y. Wu X. P. Deng X. G. Huang G. Zhu X. P. Long Z. F. (2002). Age-related bone mineral density, accumulated bone loss rate and prevalence of osteoporosis at multiple skeletal sites in chinese women. *Osteoporos. Int.* 13 669–676. 10.1007/s001980200091 12181627

[B36] Liu X. Wu W. Y. Jiang B. H. Yang M. Guo D. A. (2013). Pharmacological tools for the development of traditional Chinese medicine. *Trends Pharmacol. Sci.* 34 620–628. 10.1016/j.tips.2013.09.004 24139610

[B37] Liu X. Zhang S. Lu X. Zheng S. Li F. Xiong Z. (2012). Metabonomic study on the anti-osteoporosis effect of rhizoma drynariae and its action mechanism using ultra-performance liquid chromatography–tandem massspectrometry. *J. Ethnopharmacol.* 139 311–317. 10.1016/j.jep.2011.11.017 22120013

[B38] Lofman O. Magnusson P. Toss G. Larsson L. (2005). Common biochemical markers of bone turnover predict future bone loss: a 5-year follow-up study. *Clin. Chim. Acta* 356 67–75. 10.1016/j.cccn.2004.12.014 15936304

[B39] Ma B. Liu J. Zhang Q. Ying H. Jiye A. Sun J. (2013). Metabolomic profiles delineate signature metabolic shifts during estrogen deficiency-induced bone loss in rat by GC-TOF/MS. *PLoS One* 8 1–10. 10.1371/journal.pone.0054965 23408954 PMC3567117

[B40] Maceyka M. Harikumar K. B. Milstien S. Spiegel S. (2012). Sphingosine-1-phosphate signaling and its role in disease. *Trends Cell Biol.* 22 50–60. 10.1016/j.tcb.2011.09.003 22001186 PMC3253987

[B41] Marion D. Machuca-Gayet I. Kikuta J. Ottewell P. Mima F. Leblanc R. (2014). Lysophosphatidic acid receptor type 1 (LPA1) plays a functional role in osteoclast differentiation and bone resorption activity. *J. Biol. Chem.* 289 6551–6564 10.1074/jbc.M113.533232 24429286 PMC3945319

[B42] Matsumoto T. Kobayashi T. Kamata K. (2007). Role of lysophosphatidylcholine (LPC) in atherosclerosis. *Curr. Med. Chem.* 14 3209–3220. 10.2174/092986707782793899 18220755

[B43] Moloney M. G. (2016). Natural products as a source for novel antibiotics. *Trends pharmacol. Sci.* 37 689–701. 10.1016/j.tips.2016.05.001 27267698

[B44] Reginster J. Y. Sarkar S. Zegels B. Henrotin Y. Bruyere O. Agnusdei D. (2004). Reduction in PINP, amarker of bone metabolism, with raloxifene treatment and its relationship with vertebral fracture risk. *Bone* 34 344–351. 10.1016/j.bone.2003.10.004 14962813

[B45] Salles J. P. Laurencin-Dalicieux S. Conte-Auriol F. Briand-Mésange F. Gennero I. (2013). Bone defects in LPA receptor genetically modified mice. *Biochim. Biophys. Acta* 1831 93–98. 10.1016/j.bbalip.2012.07.018 22867754

[B46] Shi J. Cao B. Wang X. W. Aa J. Y. Duan J. A. Zhu X. X. (2016). Metabolomics and its application to the evaluation of the efficacy and toxicity of traditional Chinese herb medicines. *J. Chromatogr.* 1026 204–216. 10.1016/j.jchromb.2015 26657802

[B47] Sims S. M. Panupinthu N. Lapierre D. M. Pereverzev A. Dixon S. J. (2013). Lysophosphatidic acid: a potential mediator of osteoblast–osteoclast signaling in bone. *Biochim. Biophys. Acta* 1831 109–116. 10.1016/j.bbalip.2012.08.001 22892679

[B48] Sun P. Hu L. P. Dong Q. W. Cai M. Q. Wang X. D. Guo J. (2016). Effect of FTZ on BMD and biomechanics in the femur and lumbar vertebrae in glucocorticoid-treated rats. *Chin. J. Osteoporos.* 22 135–138.

[B49] Tilg H. Moschen A. R. Kaser A. Pines A. Dotan I. (2008). Gut, inflammation and osteoporosis: basic and clinical concepts. *Gut* 57 684–694. 10.1136/gut.2006.117382 18408105

[B50] Wang M. Chen L. Liu D. Chen H. (2017). Metabolomics highlights pharmacological bioactivity and biochemical mechanism of traditional Chinese medicine. *Chem. Biol. Interact.* 273 133–141. 10.1016/j.cbi.2017.06.011 28619388

[B51] Wang M. Chen D. Q. Chen L. Cao G. Zhao H. Liu D. (2018a). Novel inhibitors of the cellular renin-angiotensin system components, poricoic acids, target Smad3 phosphorylation and Wnt/beta-catenin pathway against renal fibrosis. *Br. J. Pharmacol.* 175 2689–2708. 10.1111/bph.14333 29679507 PMC6003649

[B52] Wang M. Chen D. Q. Chen L. Zhao H. Liu D. Zhang Z. H. (2018b). Novel RAS inhibitors poricoic acid ZG and poricoic acid ZH attenuate renal fibrosis via Wnt/β-catenin pathway and targeted phosphorylation of smad3 signaling. *J. Agric. Food Chem.* 66 1828–1842. 10.1021/acs.jafc.8b00099 29383936

[B53] Weske S. Vaidya M. Reese A. von Wnuck Lipinski K. Keul P. Bayer J. K. (2018). Targeting sphingosine-1-phosphate lyase as an anabolic therapy for bone loss. *Nat. Med.* 24 667–678 10.1038/s41591-018-0005-y 29662200

[B54] Zhang Y. Li Q. Wan H. Y. Helferich W. G. Wong M. S. (2009). Genistein and a soy extract differentially affect three-dimensional bone parameters and bone-specific gene expression in ovariectomized mice. *J. Nutr.* 139 2230–2236. 10.3945/jn.109.108399 19793844

[B55] Zhao X. Wu Z. X. Zhang Y. Yan Y. B. He Q. Cao P. C. (2011). Anti-osteoporosisactivity of *Cibotium barometz* extract on ovariectomy-induced bone loss in rats. *J. Ethnopharmacol.* 137 1083–1088. 10.1016/j.jep.2011.07.017 21782010

[B56] Zhao Y. Y. Cheng X. L. Wei F. Bai X. Tan X. J. Lin R. C. (2013). Intrarenal metabolomic investigation of chronic kidney disease and its TGF-β1 mechanism in induced-adenine rats using UPLC Q-TOF/HSMS/MSE. *J. Proteome Res.* 12 2692–2703. 10.1021/pr3007792 23227912

[B57] Zhong X. L. Guo J. Wang L. Y. Luo D. S. Bei W. J. Chen Y. Y. (2012). Analysis of the constituents of the prototypeand metabolite constituents in rat serum after oral administration of fufang zhen zhu tiao zhi capsule by UPLC-Q-TOF MS/MS. *Chromatographia* 75 111–129. 10.1007/s10337-011-2164-6 22307991 PMC3264872

