# Supplementary figures and images for: Untargeted Metabolomics Reveals the Protective Effect of Fufang Zhenshu Tiaozhi (FTZ) on Aging-Induced Osteoporosis in Mice
Source: Front Pharmacol. 2019 Jan 8;9:1483. doi: 10.3389/fphar.2018.01483 (PMC6331458; doi:10.3389/fphar.2018.01483)

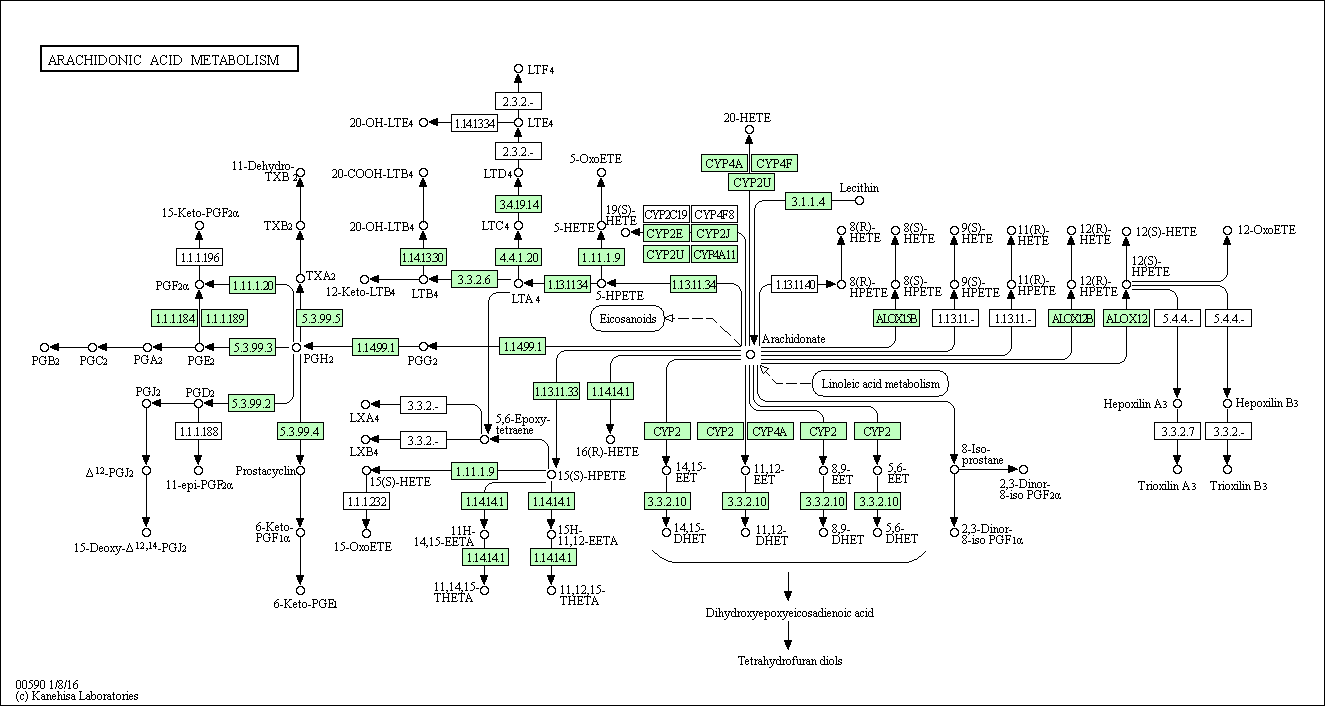

Supplement: FIGURE S1 — Overview of arachidonic acid metabolism with MetPA (reference map by KEGG). Green boxes represent enzymatic activities with putative cases of analogy in mice. [file Image_1.png]

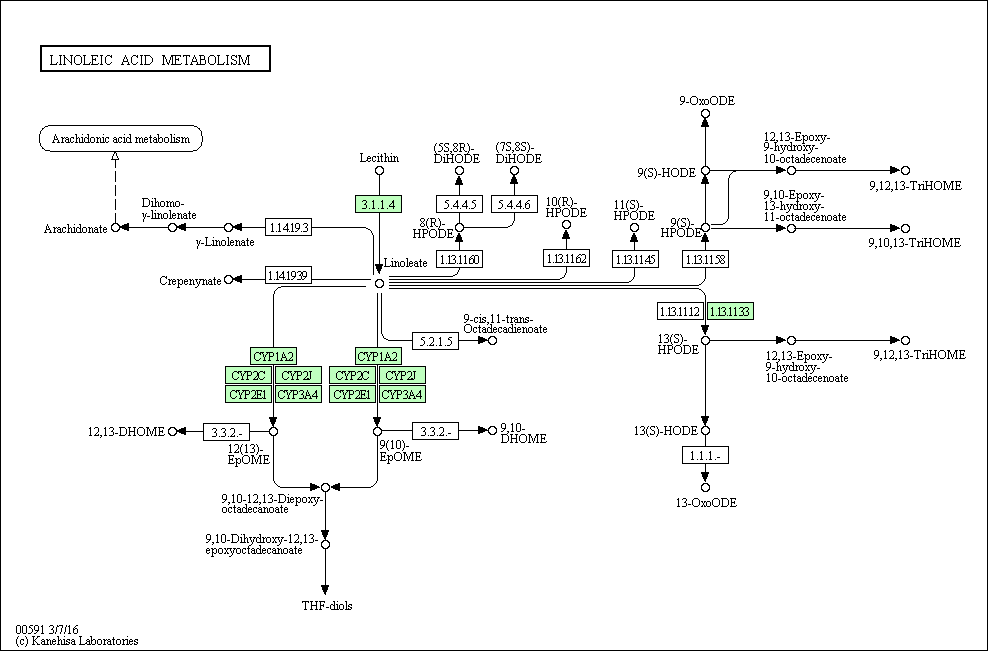

Supplement: FIGURE S2 — Overview of linoleic acid metabolism with MetPA (reference map by KEGG). Green boxes represent enzymatic activities with putative cases of analogy in mice. [file Image_2.png]

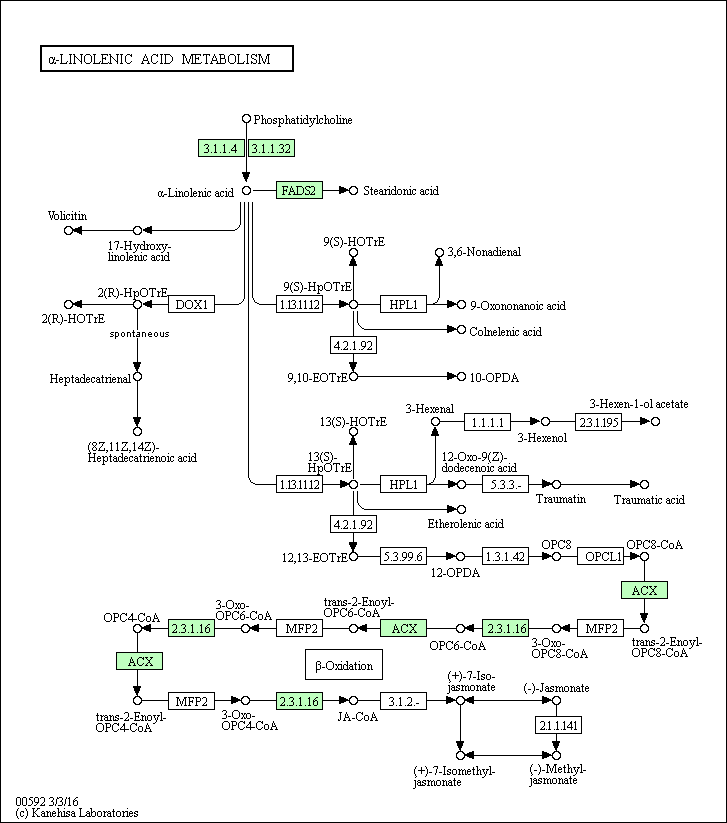

Supplement: FIGURE S3 — Overview of alpha-linolenic acid metabolism with MetPA (reference map by KEGG). Green boxes represent enzymatic activities with putative cases of analogy in mice. [file Image_3.png]

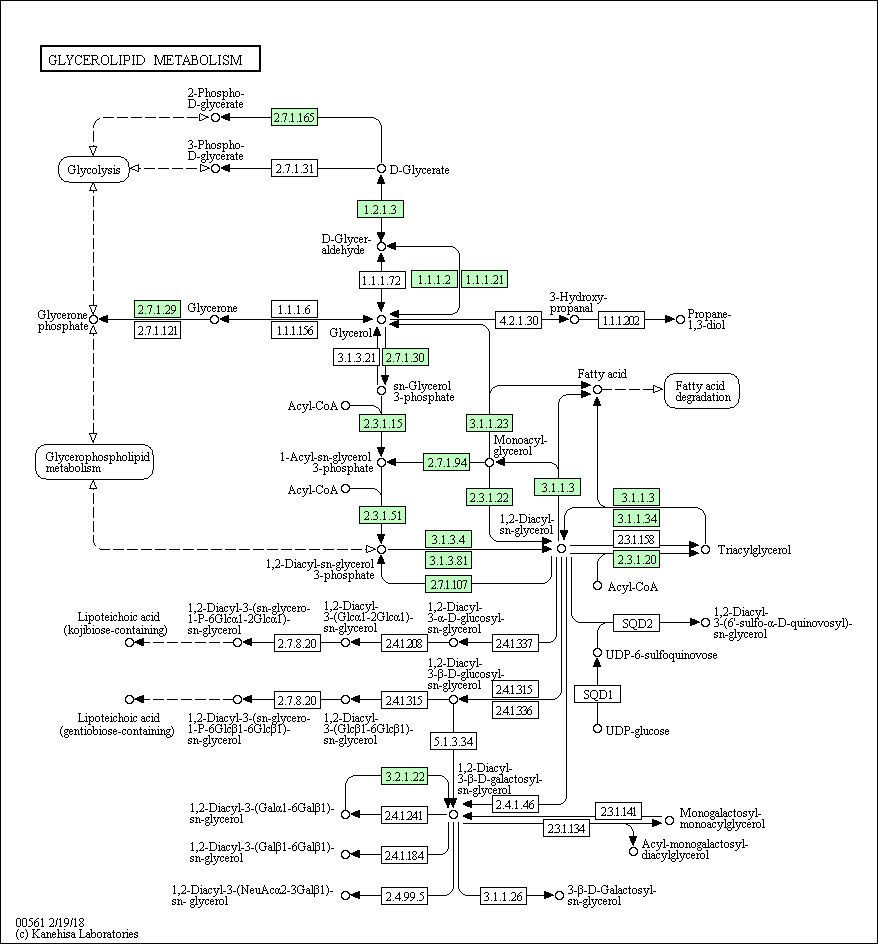

Supplement: FIGURE S4 — Overview of glycerolipid metabolism with MetPA (reference map by KEGG). Green boxes represent enzymatic activities with putative cases of analogy in mice. [file Image_4.png]

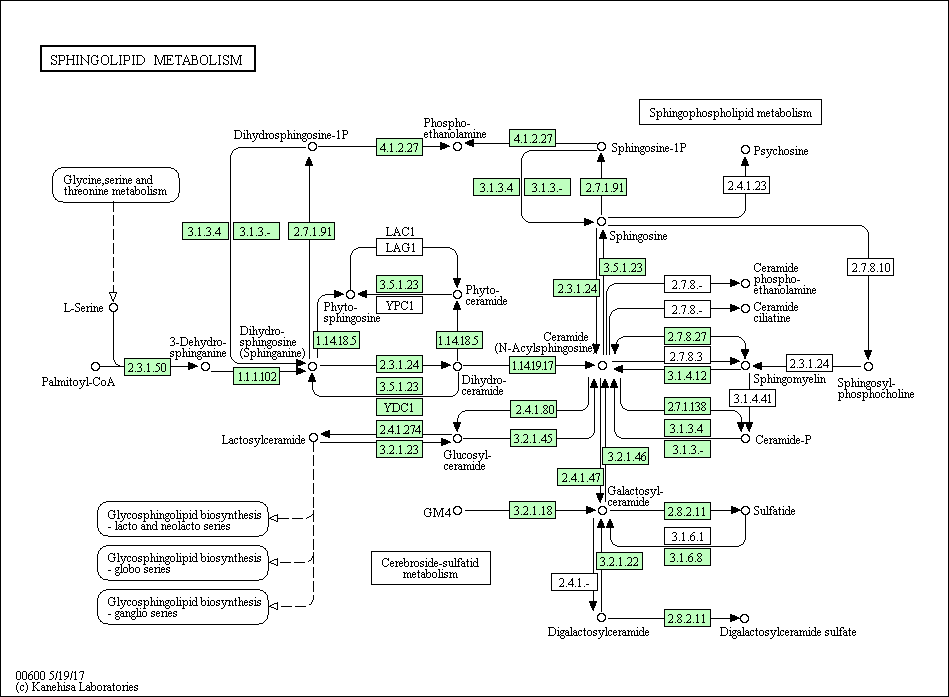

Supplement: FIGURE S5 — Overview of sphingolipid metabolism with MetPA (reference map by KEGG). Green boxes represent enzymatic activities with putative cases of analogy in mice. [file Image_5.png]

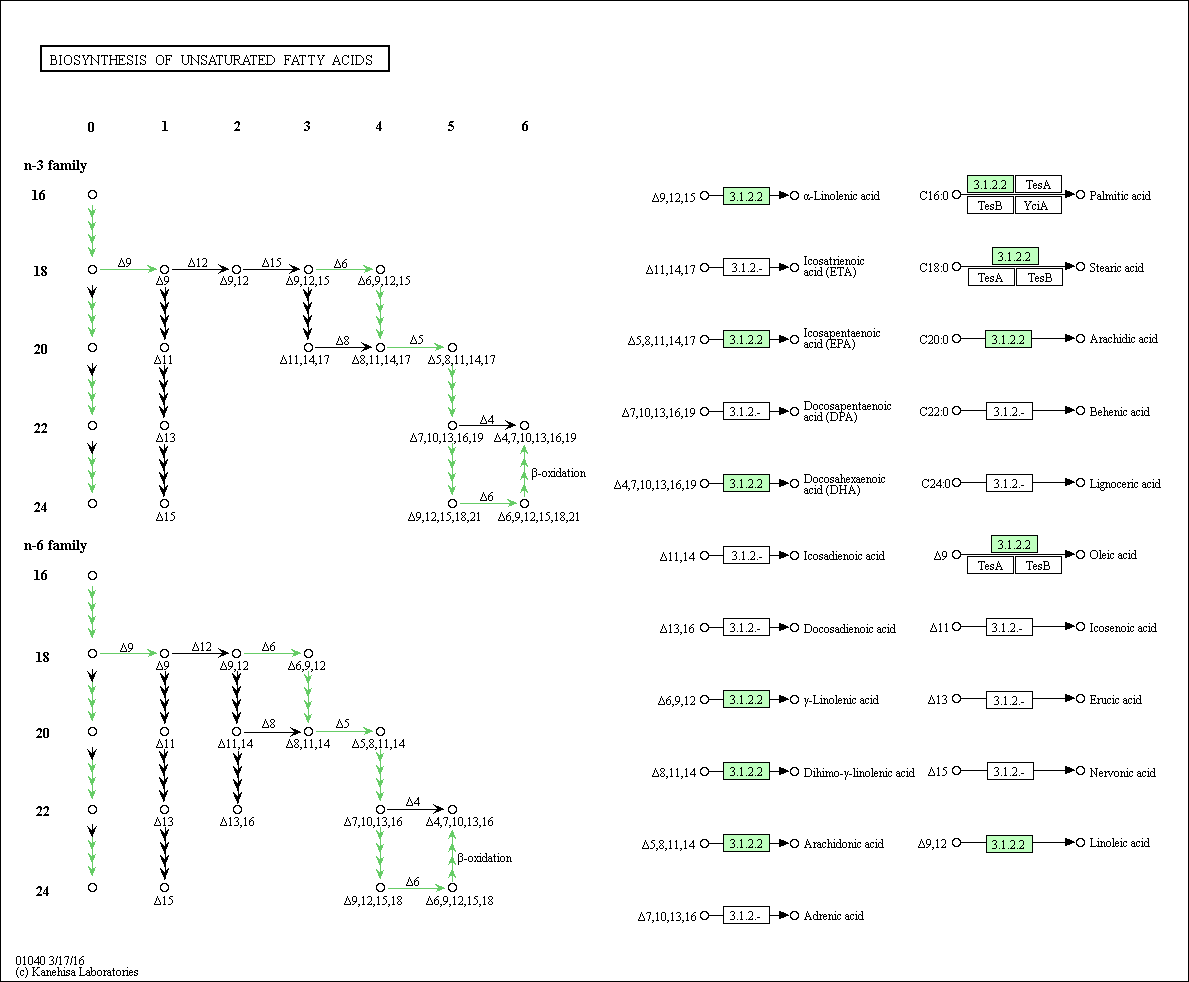

Supplement: FIGURE S6 — Overview of biosynthesis of unsaturated fatty acids with MetPA (reference map by KEGG). Green boxes represent enzymatic activities with putative cases of analogy in mice. [file Image_6.png]

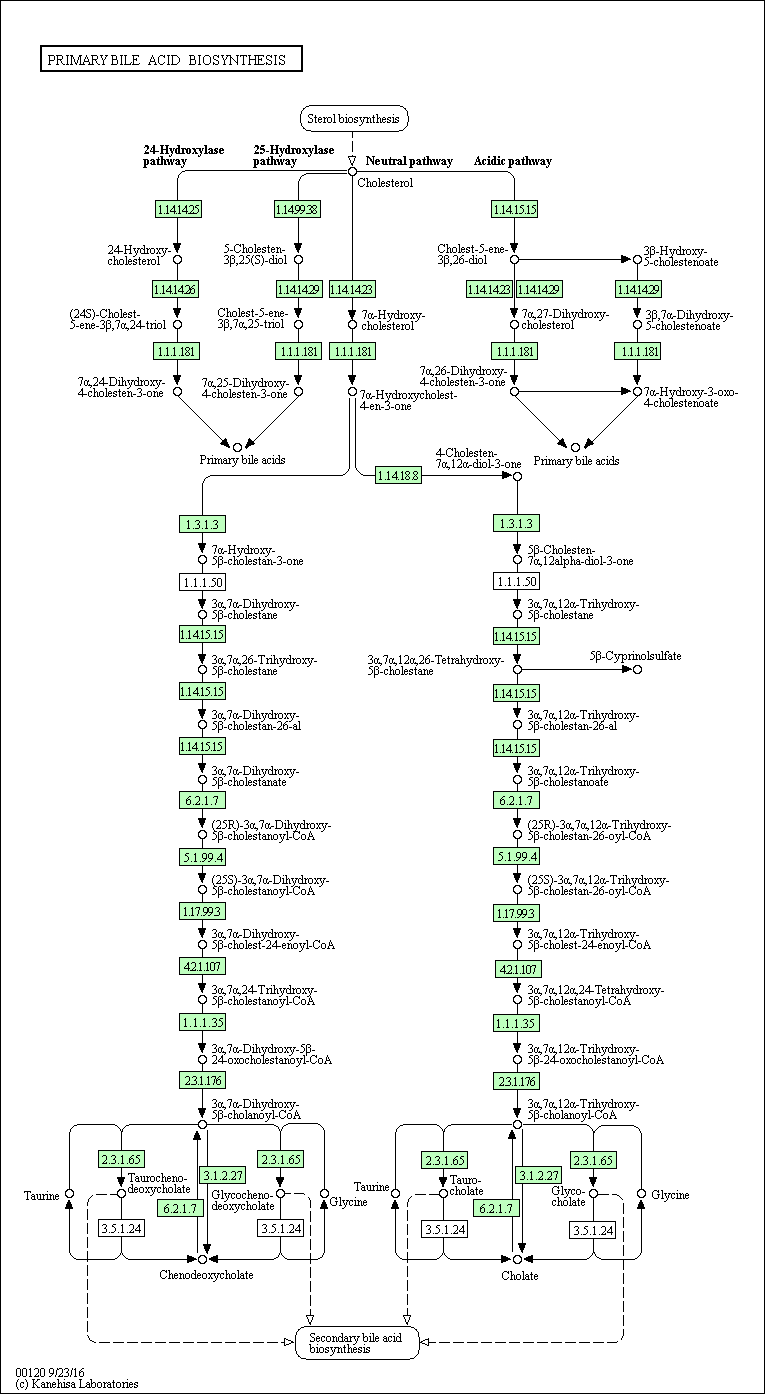

Supplement: FIGURE S7 — Overview of primary bile acid biosynthesis with MetPA (reference map by KEGG). Green boxes represent enzymatic activities with putative cases of analogy in mice. [file Image_7.png]
