# Supplementary material for: Untargeted Metabolomics Reveals the Protective Effect of Fufang Zhenshu Tiaozhi (FTZ) on Aging-Induced Osteoporosis in Mice
Source: Front Pharmacol. 2019 Jan 8;9:1483. doi: 10.3389/fphar.2018.01483 (PMC6331458; doi:10.3389/fphar.2018.01483)
Supplement: DATA SHEET S1 — Analytical system and chromatographic conditions of FTZ by HPLC. [file Data_Sheet_1.DOCX]

Analytical system and chromatographic conditions of FTZ by HPLC

A Waters 2695 Alliance HPLC system (Waters Corp.,Milford, MA, USA), equipped with EmpowerTM Software and consisted of a quaternary solvent delivery system, an on-line degasser, an autosampler, a thermostatic compartment and a 2998 diode array detector (DAD) coupled with a 2424 evaporative light scattering detectors

(ELSD), was used for the chromatographic analysis. All separations were carried out on an Ultimate XB C18 column (250 mm×4.6 mm, 5.0 μm) and the column temperature was maintained at 30 ºC. The mobile phase were composed of A (acetonitrile) and B (0.25% glacial acetic acid and 0.13% triethylamine in water, v/v). The separation was achieved using gradient elution of 0–5 min, 5% A; 5–35 min,

5–30% A; 35–45 min, 30% A; 45–55 min, 30–78% A;55–85 min, 78% A, then keeping 100% A for 15 min to wash the column. Finally the column was reconditioned with 5% A for 15 min. The flow rate was 0.8 mL/min. An aliquot of

10 μL of each sample was injected. Detection wavelengths were set at 225 nm for salidroside (**1**) and specneuzhenide (**2**),270 nm for magnoflorine (**3**), columbamine (**6**), jatrorrhizine(**7**), epiberberine (**8**), coptisine (**9**), palmatine (**10**), berberine(**11**), 286 nm for salvianolic acid B (**5**), 326 nm for rosmarinic acid (**4**) and 5,7-dimethoxycoumarin (**12**). The ginsenoside Rg1 (**13**), ginsenoside Rb1 (**14**) and oleanic acid (**15**) were

monitored using the ELSD. The ELSD parameters were set at a drift tube temperature of 80 ºC and a nitrogen gas pressure of 30 psi, with nebulizer heated at 60% power

level. Data acquisition and processing were performed with Empower software.
